# Urolithiasis and Its Treatment in Pregnant Women: 10-Year Clinical Experience From a Single Centre

**DOI:** 10.7759/cureus.13752

**Published:** 2021-03-07

**Authors:** Mehmet Demir, İsmail Yagmur, Eyyup S Pelit, Bülent Katı, Eser Ördek, Halil Çiftçi

**Affiliations:** 1 Department of Urology, Harran University, Şanlıurfa, TUR

**Keywords:** urolithiasis, kidney stones, health, pregnancy

## Abstract

Introduction: Urolithiasis during pregnancy is an important health concern that can affect maternal and foetal health. If left untreated, it can cause obstetric complications, such as spontaneous abortion and preterm delivery. In this study, we aimed to evaluate urolithiasis and its treatment in pregnant women.

Methods: We analysed data of 57 patients diagnosed with urolithiasis during pregnancy between January 2010 and December 2020. Patients’ age, gestational age, urolithiasis history, physical examination findings, laboratory findings, location and size of the stone and applied treatment methods were examined. The effectiveness and complications of the applied treatment methods were evaluated.

Results: The mean age of 57 patients included in our study was 27 (27.8 ± 5.6) years and their mean gestational age was 20 (20.3 ± 9.2) weeks. The mean stone size was 9 mm (9.09 ± 4.37). The most common symptom at the time of presentation to the hospital was flank pain (93%). Kidney stones were detected in 34 patients and ureteral stones in 23 patients. Although conservative treatment was sufficient in 26 (45.6%) patients, 31 (54.4%) patients required surgical intervention. Major obstetric complications, such as preterm delivery and abortion, did not occur in any of the patients.

Conclusion: The diagnosis and treatment of pregnant women with urolithiasis should be managed in a multidisciplinary manner. While determining the treatment options, foetal and maternal health should be considered.

## Introduction

Urolithiasis during pregnancy is an important health problem that can affect the maternal and foetal health [1]. In previous studies, the incidence of urolithiasis during pregnancy has been reported to be between 1:188 and 1:4600. Studies evaluating the effect of geographical location show that the incidence of urolithiasis is increasing in industrialised societies. However, there is minimal data about the role of geographical location in the incidence and prevalence of urolithiasis in pregnant women [1,2]. Urolithiasis may lead to urinary stasis, causing obstetric complications, such as preterm delivery and spontaneous abortion associated with urinary tract infection and pyelonephritis in pregnant women [3]. The diagnosis of urolithiasis during pregnancy poses a number of difficulties owing to the limitations in the use of imaging methods. The differential diagnoses in pregnant women presenting with complaint of flank pain are usually urolithiasis as well as pregnancy-related physiological hydronephrosis. Urolithiasis during pregnancy may mimic other emergencies, such as appendicitis and diverticulitis, and its diagnosis may be delayed [4]. Thus, 80%-90% of the patients are diagnosed in the second and third trimesters [5]. Conservative treatment is the first option in the treatment of urolithiasis, as approximately 80% of the stones can be passed spontaneously during pregnancy. However, 20%-30% of patients may require surgical intervention [6]. In this study, we aimed to evaluate the results of patients we treated for urolithiasis during pregnancy.

## Materials and methods

For this study, we retrospectively reviewed the hospital records of pregnant women who presented to the Harran University Hospital Urology Clinic with complaints of flank pain between January 2010 and December 2020 after obtaining approval from the ethics committee of our faculty (HRU/21.01.17). Among these patients, those who were diagnosed with urolithiasis were included in this study. Patient’s age, gestational age, trimester, urolithiasis history, physical examination findings, serum creatinine level, complete blood count, complete urine analysis, urine culture, location and size of the stone and treatment method were recorded.

Urolithiasis was diagnosed by the evaluation of the clinical findings of the patients, presence of microscopic haematuria in urinalysis, ultrasound (US) findings and ureteroscopy findings. X-ray imaging and computed tomography (CT) were not used. Medical treatment comprised fluid therapy, safe analgesics and antibiotic treatment according to the antibiogram if infection was present. Surgical treatment comprised percutaneous nephrostomy, ureteral stenting and ureteroscopy as deemed appropriate for the patient. Percutaneous nephrostomy was performed with US guidance and under local anaesthesia, and a ureteral stent was inserted under local anaesthesia or sedoanalgesia. Percutaneous nephrostomy was performed in the lateral position. Ureteroscopy was performed under general or spinal anaesthesia after meeting the conditions for sterile urine culture in patients scheduled for definitive treatment. Ureteroscopy was performed with a 4.5 F or 6.9 F semirigid ureteroscope (Karl Storz, Germany) under direct vision. Stones below the iliac artery pulsation were classified as distal ureteral stones and those above as proximal ureteral stones. The stones were fragmented with holmium:yttrium-aluminum-garnet (YAG) laser or pneumatic lithotripter. All patients who underwent surgical treatment were referred to the gynaecology department for obstetric evaluation in the early postoperative period.

Statistical method

Mean, standard deviation, median, lowest, highest, frequency and ratio values were used in the descriptive statistics of the data. SPSS 27.0 (IBM Corp., Armonk, NY) program was used in the analyses.

## Results

The mean age of 57 patients diagnosed with and treated for urolithiasis during pregnancy in our clinic was 27 (27.8 ± 5.6) years and their gestational age was 20 (20.3 ± 9.2) weeks.The mean stone size was 9 mm (9.09 ± 4.37) (Table 1). Although 30 of the patients had a history of urolithiasis before pregnancy, 27 of them had no history of urolithiasis. The most common reason for presenting to the hospital was flank pain (93%). Fever was detected in 15 patients and pyelonephritis in 8 patients at presentation. Microscopic haematuria was detected in 54.4% and pyuria in 57.9% of the patients in complete urinalysis (Table 2). Kidney stones were detected in 34 patients and ureteral stones in 23 patients. Stone locations are detailed in Table 3. Conservative treatment was sufficient in 26 patients (45.6%), whereas 31 patients (54.4%) required surgical intervention (Table 4). Major obstetric complications such as preterm delivery and abortion were not observed in any patients.

**Table 1:** Patient demographics

	Min-Max	Mean ± SD
Age (years)	18.0-43.0	27.8 ± 5.6
Gestation weeks	4.0-40.0	20.3 ± 9.2
Creatinine (mg/dl)	0.4-1.3	0.7 ± 0.2
Stone size (mm)	1.5-30.0	9.1 ± 4.4

 

**Table 2:** Clinical and laboratory findings of patients

		n	%
Trimester	I	15	26.3%
	II	28	49.1%
	III	14	24.6%
Symptoms	Flank pain	53	93.0%
	Dysuria	4	7.0%
Stone side	Bilateral	5	8.8%
	Right	24	42.1%
	Left	28	49.1%
Fever	Yes	15	26.3%
	No	42	73.7%
Pyelonephritis	Yes	8	14.0%
	No	49	86.0%
History of urolithiasis	Yes	30	52.6%
	No	27	47.4%
Microscopic pyuria	Yes	33	57.9%
	No	24	42.1%
Microscopic hematuria	Yes	31	54.4%
	No	26	45.6%
Positive urine culture	Yes	17	29.8%
	No	40	70.2%

**Table 3:** Distribution of locations of stones in the urinary system

Stone location	n	%
Renal pelvis	23	37%
Upper pole	4	6.5%
Middle pole	3	4.8%
Lower pole	7	11.3%
Staghorn	2	3.2%
Proximal ureter	12	19.4%
Distal ureter	11	17.7%

**Table 4:** Distribution of the treatment methods applied to the patients

Treatment	n	%
Medical therapy	26	45.6%
Ureteroscopy	18	31.6%
Double-J stent insertion	11	19.3%
Percutaneous nephrostomy	2	3.5%

## Discussion

The most common non-obstetric cause of abdominal pain requiring hospitalisation in pregnant women is flank pain due to urolithiasis [7]. Although 85%-100% of patients present with flank pain, there may also be accompanying lower urinary tract symptoms or haematuria. In the studies performed, microscopic haematuria was reported at a rate between 95% and 100% on repeated urinalysis examinations [8]. In our study, it was found that 93% of the patients presented to the hospital with flank pain and 7% with lower urinary tract symptoms. Urinalysis revealed microscopic haematuria in 54.4% of the patients at presentation.

Urolithiasis can lead to urinary stasis, causing urinary tract infection and pyelonephritis. Consequently, obstetric complications may develop in pregnant women [3,9]. In a study conducted by Stothers and Lee, the incidence of pyuria was reported to be 42% and urine culture positivity 24% [10]. In our study, the incidence of pyuria was found to be 57.9%, whereas the rate of positive urine culture was 29.8%. Simultaneous pyelonephritis was detected in 14% of the patients at presentation.

Owing to the limitations in the use of imaging methods during pregnancy, there may be delays in diagnosis [4]. Therefore, 80%-90% of the patients are diagnosed in the second and third trimesters [5]. In this study, 26.3% of the patients were diagnosed in the first trimester and 73.7% in the second and third trimesters. Although CT is the gold standard diagnostic method in the evaluation of renal colic in adults, it is not a preferred diagnostic method in pregnant women due to its potential teratogenic effect [11]. Although US has lower specificity than CT in the diagnosis of urolithiasis, it is a harmless method for the mother and foetus and can be repeated. Therefore, it is the first choice for imaging in pregnant women presenting with renal colic [12]. Recent studies have shown that a radiation dose lower than 50 mGy is not associated with a high risk of malformation in pregnant women [11,13]. Therefore, it has been reported that reasonable use of low-dose CT scanning protocols may be an option in the diagnosis of renal colic [14,15]. In our study, the diagnosis of urolithiasis was made based on the clinical findings, urinalysis findings and US findings, and CT was not performed in any patient.

Urolithiasis can cause hydronephrosis and may be confused with pregnancy hydronephrosis. Pregnancy hydronephrosis is mostly asymptomatic [16]. In symptomatic patients, the most commonly reported clinical symptom is flank pain, similar to that in urolithiasis [10]. Gestational hydronephrosis occurs two to three times more frequently on the right side than the left side [17], whereas urolithiasis occurs at similar rates on both sides [8]. In addition, pregnant women with urolithiasis may have a previous history of stones. In previous studies, this rate was reported to be between 24% and 30% [10,18]. In our study, 42.1% of the patients had right, 49.1% left and 8.8% bilateral urolithiasis. Of these patients, 52.6% had a history of urolithiasis. Pregnant women presenting with flank pain are more likely to have urolithiasis if the pain is on the left side and there is a history of stones. Utmost care should be taken when evaluating such patients.

Medical treatment is recommended as the first option in the treatment of urolithiasis in pregnant women. Medical treatment includes intravenous fluid, safe analgesics and in the presence of infection, antibiotic treatment according to an antibiogram. In the literature, it has been reported that 50%-84% of the stones are spontaneously passed with medical treatment [7,8]. In the present study, response to medical treatment was achieved in 45.6% of the patients, which was lower than that in previous studies. Because our hospital is a highest level referral centre, patients who require surgery are more often referred to our hospital. This may be the reason why the rate of response to medical therapy was low in this study. Surgical intervention is required in 20%-30% of pregnant women with urolithiasis [6]. Surgical treatment is recommended in the case of uncontrolled pain, persistent vomiting, fever, obstetric complications, solitary kidney and bilateral ureteral stones. In surgical treatment, temporary drainage can be achieved with percutaneous nephrostomy and ureteral JJ stent, and definitive treatment can also be performed with ureteroscopy [1,7,8]. Percutaneous nephrolithotomy and extracorporeal shockwave lithotripsy are contraindicated during pregnancy according to the current guidelines due to high complication rates and teratogenic risk [19]. Percutaneous nephrostomy is a US-guided drainage method performed under local anaesthesia and has a success rate of >90%. It has advantages, such as being a minimally invasive method, providing acute and effective drainage in patients with sepsis, being applied under local anaesthesia and causing no radiation exposure [20-22]. In our study, percutaneous nephrostomy catheter was inserted in two patients who presented with sepsis findings and were found to have pyonephrosis. There were no complications in patients who were administered appropriate antibiotic therapy and supportive treatment. Definitive treatment of the patients was performed after pregnancy.

Ureteral JJ stent is an effective method enabling acute drainage that can be inserted under local anaesthesia or sedation in pregnant women. However, it has disadvantages, such as being a temporary treatment, needing to be changed periodically and causing irritative lower urinary tract symptoms [23,24]. In this study, a ureteral JJ stent was inserted in 11 patients to provide drainage, and no obstetric complications occurred in such patients. Irritative lower urinary tract symptoms occurred in four patients. The JJ stent was changed every three months until the pregnancy terminated.

Ureteroscopy, which is safe in all trimesters, is used in the definitive treatment of ureteral stones in pregnant women who do not respond to medical treatment [25]. In ureteroscopy, pneumatic lithotripter and holmium:YAG laser are commonly used for stone fragmentation [26]. Pneumatic lithotripter and holmium:YAG laser were compared for stone fragmentation in a study by Bozkurt et al., and it has been reported that both methods are safe in pregnancy [25]. Tissue penetration is lower in holmium:YAG laser when compared with pneumatic lithotripter. Therefore, theoretically it poses a lower risk for foetal injury. In addition, stone migration is less common in holmium:YAG laser [5]. For stone fragmentation, holmium:YAG laser was used in 16 and pneumatic lithotripter was used in 2 patients who were diagnosed with ureteral stones and underwent definitive ureteroscopy in our study. No obstetric complications occurred with either of the method.

## Conclusions

Urolithiasis can occur in pregnant women and its diagnosis can be difficult. Therefore, a multidisciplinary approach should be undertaken for evaluation, accurate diagnosis and treatment of urolithiasis. In determining the treatment options, foetal and maternal health should be considered. In cases where surgical intervention is required, the treatment method should be chosen based on the clinical findings, location of the stone, available facilities and experience of the surgeon. Percutaneous nephrostomy, ureteral stent and ureteroscopy can be used safely in all trimesters at experienced centres.
